# A Minor Sports Injury with Major Consequences: Probable Streptococcal Toxic Shock Syndrome and Necrotizing Soft Tissue Infection in a Young Adult—A Case Report

**DOI:** 10.3390/reports9030215

**Published:** 2026-07-09

**Authors:** Bartosz Stangiewicz, Lukasz Korzep

**Affiliations:** 1 Our Lady of Perpetual Help Hospital, 05-200 Wołomin, Poland; lukasz.korzep@szpitalwolomin.pl; 2 Department of Clinical Dietetics, Faculty of Health Sciences, Medical University of Warsaw, 01-445 Warsaw, Poland

**Keywords:** *Streptococcus pyogenes*, streptococcal toxic shock syndrome, necrotizing soft tissue infection, blunt trauma, continuous renal replacement therapy

## Abstract

**Background and Clinical Significance:** *Streptococcus pyogenes* (group A *Streptococcus*, GAS) can cause rapidly progressive invasive infections, including necrotizing soft tissue infection (NSTI) and streptococcal toxic shock syndrome (STSS). Although invasive GAS disease is often associated with skin barrier disruption, severe infection may also follow blunt trauma without visible skin injury. **Case Presentation:** A 22-year-old woman presented with persistent right hip and groin pain four days after a blunt fall during recreational sports activity, without disruption of skin integrity. On admission, she was hypotensive, tachycardic, and intermittently hypoxemic, with local hematoma, swelling, and inflammatory infiltration of the right groin. Laboratory tests showed marked inflammation, acidosis, acute kidney injury (AKI), elevated lactate, creatine kinase, and myoglobin levels. She was admitted to the intensive care unit with septic shock. Empirical antimicrobial therapy was initiated with piperacillin/tazobactam, clindamycin, and linezolid. Computed tomography showed inflammatory changes extending from the right groin to the thigh fascia. On day 3, the patient’s condition deteriorated with respiratory failure necessitating endotracheal intubation and mechanical ventilation. Surgical incision revealed inflamed and necrotic subcutaneous tissue with superficial muscle involvement. Deep tissue cultures yielded GAS, whereas blood and urine cultures remained negative; probable STSS was diagnosed. Therapy was de-escalated to penicillin plus clindamycin. Continuous renal replacement therapy with an adsorptive acrylonitrile 69 surface-treated (AN69ST) membrane was initiated for AKI. The patient gradually improved and was transferred to the surgical ward on day 16. **Conclusions:** Minor blunt trauma without skin disruption may precede life-threatening invasive GAS infection. Rapid recognition, surgical source control, antitoxin antimicrobial therapy, and intensive organ support are essential in suspected STSS.

## 1. Introduction and Clinical Significance

*Streptococcus pyogenes* (group A *Streptococcus*, GAS) is an important human pathogen responsible for a broad spectrum of diseases, ranging from common and usually mild infections, such as pharyngitis, scarlet fever, and superficial skin infections, to severe invasive infections, including NSTI, bacteremia, and streptococcal toxic shock syndrome (STSS) [1,2]. Invasive GAS infections may follow a rapidly progressive course, sometimes without an identifiable portal of entry, leading to septic shock, multiorgan failure, and death. STSS is among the most severe manifestations of *S. pyogenes* infection; in the European Strep-EURO study, the 7-day case-fatality rate for severe *S. pyogenes* disease was 19%, increasing to 44% among patients with STSS [3]. According to the Centers for Disease Control and Prevention/Council of State and Territorial Epidemiologists (CDC/CSTE) definition, STSS is associated with invasive or non-invasive GAS infection and is characterized by systemic toxicity and a rapidly progressive clinical course. Diagnostic criteria include isolation of GAS, hypotension, and at least two manifestations of organ or systemic involvement [4]. The pathophysiology of STSS is largely toxin-mediated: exotoxins, superantigens, and other virulence factors of *S. pyogenes* may induce massive immune activation and a cytokine cascade, leading to shock and organ dysfunction [5,6].

The management of STSS requires simultaneous interventions in several areas: early recognition and treatment of septic shock, prompt initiation of antimicrobial therapy, and urgent source control [5,7]. In cases complicated by NSTI, aggressive surgical management, including early exploration and debridement of necrotic tissues, is of fundamental importance [8]. Once group A streptococcal infection is suspected or confirmed, penicillin combined with clindamycin remains the treatment of choice. Clindamycin serves as an antitoxin agent by inhibiting bacterial protein synthesis, including the production of streptococcal toxins [5,8]. Intensive supportive care is also required, including fluid resuscitation, vasopressors, mechanical ventilation, and renal replacement therapy when indicated [7]. Intravenous immunoglobulins (IVIGs) may be considered as adjunctive therapy in severe STSS because of their potential toxin-neutralizing effect; however, the quality of available evidence remains limited, and their routine use is not clearly established as a standard of care [5,8,9,10].

We present the case of a 22-year-old woman who developed *S. pyogenes* NSTI with clinical features consistent with probable STSS after a seemingly minor blunt injury to the lower limb, without disruption of skin integrity. This case illustrates the fulminant course of invasive GAS infection and highlights the importance of early recognition, source control, antitoxin therapy, and intensive supportive care.

## 2. Case Presentation

A 22-year-old woman presented to the emergency department because of pain in the right hip and groin region, which had developed after a fall during recreational sports activity four days earlier. Her medical history included epilepsy, anxiety disorder, and insulin resistance. The injury was blunt, with no disruption of skin integrity.

On admission, the patient was conscious, alert, and able to communicate appropriately. She reported persistent pain in the right groin, generalized weakness, dizziness, and nausea. Physical examination revealed a hematoma, swelling, and increased local warmth in the right groin region, with inflammatory infiltration extending to the right labium majus. The pain was severe and appeared disproportionate to the initially limited external findings. The patient was hypotensive, with a blood pressure of 66/35 mmHg, tachycardic, with a heart rate of up to 160 beats/min, and had intermittent oxygen desaturation to 88%, requiring oxygen supplementation via a face mask.

Laboratory tests performed on admission showed elevated inflammatory markers, acidosis, features of AKI, increased creatine kinase and myoglobin levels, and elevated lactate concentration (Table 1). In the emergency department, fluid resuscitation with crystalloid boluses was initiated; however, this did not result in a significant improvement in mean arterial pressure, and a norepinephrine infusion was started. Due to the clinical picture of septic shock, the patient was admitted to the intensive care unit (ICU).

On ICU admission, the Acute Physiology and Chronic Health Evaluation II (APACHE II) score was 19 points, the Sequential Organ Failure Assessment (SOFA) score was 6 points, and the modified Nutrition Risk in the Critically Ill (mNUTRIC) score was 3 points. Bioelectrical impedance analysis (BIA) was also performed, showing a phase angle of 3.65°. Blood and urine samples were collected for microbiological testing. Treatment initiated in the emergency department was continued, including vasopressor support. Because of anuria, diuretic therapy was initiated. Empirical antimicrobial therapy was initiated with piperacillin/tazobactam 4.5 g every 6 h, administered as prolonged infusions, clindamycin 900 mg every 8 h, and linezolid 600 mg every 12 h. The patient remained conscious and breathed spontaneously but required oxygen therapy via a simple face mask.

Computed tomography (CT) revealed increased density of the adipose tissue in the right groin region, inflammatory changes around the iliopsoas muscle, extending to the posteromedial surface of the right thigh, and the fascia of the upper thigh (Figure 1). After the initial CT examination, the patient underwent surgical consultation. At that time, conservative management with close observation was recommended because there were no unequivocal external signs of advanced soft tissue necrosis requiring immediate operative exploration. However, early NSTI could not be excluded given the disproportionate pain, septic shock at presentation, markedly elevated inflammatory markers, elevated lactate concentration, AKI, and CT findings showing inflammatory changes extending along the right groin, thigh, and fascial planes. The retrospectively calculated Laboratory Risk Indicator for Necrotizing Fasciitis (LRINEC) score was 11, supporting a high risk of NSTI; however, this score was interpreted as an adjunctive tool and not as a stand-alone diagnostic criterion.

Over the following two days, the patient’s condition remained relatively stable, although she continued to require vasopressor support. Laboratory tests showed decreasing procalcitonin and lactate levels and improvement in renal parameters. At the same time, C-reactive protein (CRP) remained markedly elevated, leukocytosis increased, and liver enzyme levels rose. Blood and urine cultures remained negative. The local inflammatory changes were assessed repeatedly during bedside examinations. The initially observed swelling, hematoma, and erythema in the right groin region gradually extended toward the right labium majus and the proximal medial thigh. Despite partial biochemical improvement, the persistence of severe pain, increasing leukocytosis, and progression of the local soft tissue changes prompted repeat surgical evaluation. Clinical photographs documenting the early progression of the lesion were not available; therefore, progression was described based on repeated clinical examinations documented in the medical records.

On the third day of hospitalization, the patient’s clinical condition suddenly deteriorated, with the development of respiratory failure and desaturation to 85%. She was intubated, and mechanical ventilation was initiated with a fraction of inspired oxygen (FiO_2_) of 1.0, which was subsequently reduced according to arterial blood gas results. Due to the progression of the local lesions, a repeat surgical consultation was requested. This time, exploration of the right groin region was performed. The procedure consisted of incision and debridement of macroscopically necrotic subcutaneous tissue; superficial muscle involvement was noted intraoperatively. No clear macroscopic fascial involvement was documented. Necrotic tissue was removed until macroscopically viable tissue was identified. Deep tissue samples from the involved infected tissues were collected intraoperatively for microbiological testing. No repeat surgical debridement was required.

On the following day, due to AKI and hemodynamic instability, continuous renal replacement therapy (CRRT) was initiated in continuous venovenous hemodiafiltration (CVVHDF) mode using a heparin-coated AN69ST membrane with adsorptive properties. Group A *Streptococcus* (*Streptococcus pyogenes*) was isolated from intraoperative deep tissue samples using standard microbiological culture methods on routine culture media and identified according to routine diagnostic procedures used in the hospital microbiology laboratory. Based on antimicrobial susceptibility results interpreted according to European Committee on Antimicrobial Susceptibility Testing (EUCAST) criteria (Table 2), antimicrobial therapy was de-escalated to penicillin 2 million units every 4 h, administered as prolonged infusions, in combination with clindamycin as before. Based on the clinical picture, fulfillment of CDC/CSTE criteria, and isolation of GAS from a non-sterile site, probable STSS was diagnosed. The CDC/CSTE clinical criteria fulfilled in this case included hypotension requiring vasopressor support, renal impairment, liver involvement, and soft tissue necrosis. Because GAS was isolated from deep tissue material obtained from the involved soft tissues, whereas blood cultures remained negative, the case was classified as probable rather than confirmed STSS.

Over the following days, a gradual improvement in the patient’s clinical condition and laboratory parameters was observed. Wound dressings were changed daily. After three days, CRRT was discontinued; sedation was subsequently reduced, and the patient was extubated on the same day. On the eighth day of hospitalization, the surgical team decided to initiate negative-pressure wound therapy. The patient’s clinical condition continued to improve; vasopressor doses were gradually reduced until complete discontinuation, diuretic therapy was stopped, and clindamycin therapy was completed after 12 days. On the 16th day of hospitalization, the patient was transferred to the surgical ward for continued wound management. Penicillin therapy was continued until hospital day 21. At the time of transfer, she was in fair general condition, hemodynamically stable, breathing spontaneously without respiratory support, and showed marked improvement in laboratory parameters.

Following transfer to the surgical ward, negative-pressure wound therapy was continued for an additional 18 days. The wound showed gradual healing, and the patient remained in good general condition, with recovery of independent ambulation. Serial laboratory tests showed no renewed increase in inflammatory markers and no deterioration of renal function. She was discharged home on hospital day 36.

## 3. Discussion

In the present case, a seemingly minor blunt injury to the lower limb, without disruption of skin integrity, preceded the development of NSTI and clinical features consistent with probable STSS. Thigh contusion and muscle tear may have created local conditions favoring bacterial seeding, such as hematoma formation, edema, microvascular injury, and relative tissue hypoxia. Although soft tissue infections are classically associated with disruption of the skin barrier, invasive GAS infections have also been reported after blunt trauma without visible skin injury [11,12]. In such cases, a possible mechanism is transient bacteremia or dissemination from a site of colonization, as *Streptococcus pyogenes* may colonize the pharynx and skin, and less severe GAS infections may, in rare cases, precede the development of STSS [13]. It should be emphasized, however, that the portal of entry often remains unclear. Therefore, even a minor injury without a skin wound, when accompanied by progressive pain, systemic features, or rapid progression of symptoms, should prompt heightened clinical suspicion for invasive GAS infection [14].

NSTI and STSS are among the most severe manifestations of bacterial infection and may rapidly lead to septic shock, multiorgan failure, and death. NSTIs represent a heterogeneous group of infections involving the skin, subcutaneous tissue, fascia, and muscles; they may be polymicrobial or monomicrobial in etiology, including infections caused by GAS, *Staphylococcus aureus*, anaerobic bacteria, and Gram-negative bacteria [8]. Toxic shock syndrome is a toxin-mediated disease classically associated with toxigenic strains of *S. aureus* or *S. pyogenes* [15]. In STSS, streptococcal exotoxins and superantigens play a key role by inducing non-specific T-cell activation and a massive cytokine response, leading to shock and organ dysfunction [5,6]. Compared with staphylococcal TSS, STSS is generally characterized by a more fulminant clinical course, a more frequent association with invasive soft-tissue infection, and higher mortality [14,15]. Importantly, the present case also illustrates that NSTI may be present at an early stage despite initially limited cutaneous findings, and that serial assessment of lesion boundaries and repeated surgical evaluation are essential when systemic toxicity persists or local findings progress. This case also underlines the importance of a multidisciplinary approach in severe NSTIs, involving intensivists, surgeons, microbiologists, infectious disease specialists, radiologists, wound care teams, and rehabilitation specialists. Such coordinated management is essential because treatment requires simultaneous source control, appropriate antimicrobial therapy, organ support, repeated reassessment, and long-term wound care [16].

The diagnosis of STSS is based on the 2010 CDC/CSTE case definition, which includes both clinical and microbiological criteria. The clinical criteria include hypotension and at least two manifestations of organ or systemic involvement: renal impairment, coagulopathy, liver involvement, acute respiratory distress syndrome (ARDS), generalized erythematous rash, or soft tissue necrosis. A case is classified as confirmed when GAS is isolated from a normally sterile site, whereas it is classified as probable when the clinical criteria are met, no other etiology is identified, and GAS is isolated from a non-sterile site [4]. In our patient, hypotension requiring vasopressor support was present, along with at least three CDC/CSTE criteria of organ or systemic involvement: renal impairment, liver involvement, and NSTI. In addition, the disease course was complicated by respiratory failure requiring mechanical ventilation and extensive local inflammatory soft-tissue changes. Although the microbiological sample was obtained intraoperatively from deep tissues involved in the inflammatory process rather than from a superficial swab, the case was classified as probable STSS because blood cultures remained negative and the CDC/CSTE definition requires isolation of GAS from a normally sterile site. Therefore, to avoid overclassification, the term probable STSS was retained [4,14].

An additional exploratory aspect of the ICU admission assessment was bioelectrical impedance analysis. The measured phase angle was 3.65°, which is low for a young adult and may have reflected severe systemic inflammation, fluid shifts, and impaired cellular integrity during septic shock [17,18,19]. However, this finding did not directly guide therapeutic decisions in the acute phase. It should therefore be interpreted as a hypothesis-generating observation rather than a validated marker for decision-making in STSS. Further studies would be needed to determine whether BIA-derived parameters have prognostic or therapeutic value in invasive GAS infections.

One of the fundamental components of STSS treatment is the rapid initiation of effective antimicrobial therapy. When GAS infection is suspected or confirmed, clindamycin plays a particularly important role, as it inhibits bacterial protein synthesis, thereby reducing the production of streptococcal toxins and virulence factors [8]. The addition of clindamycin to beta-lactam therapy is recommended for severe invasive GAS infections and is supported by observational studies in which its use was associated with lower mortality [20]. Once GAS etiology is confirmed, penicillin in combination with clindamycin remains the treatment of choice, as *S. pyogenes* remains highly susceptible to beta-lactams and naturally occurring penicillin-resistant strains have not been confirmed to date [21,22]. In our case, the initial regimen of piperacillin/tazobactam, clindamycin, and linezolid was chosen to provide broad coverage for severe sepsis with suspected NSTI, including Gram-positive, Gram-negative, and anaerobic pathogens. Clindamycin and linezolid were included because of their ability to inhibit bacterial protein synthesis and potentially reduce toxin production. After *S. pyogenes* was isolated from tissue material obtained from the inflamed area, antimicrobial therapy was de-escalated to penicillin administered as a prolonged infusion in combination with clindamycin. A continuous or prolonged beta-lactam infusion strategy may be pharmacokinetically justified in critically ill patients, as the efficacy of this drug class depends on the time during which the free drug concentration remains above the minimum inhibitory concentration (MIC) [23,24].

IVIG is one of the adjunctive therapies considered in severe STSS. Their potential mechanism of action includes neutralization of streptococcal toxins and superantigens and attenuation of the toxin-mediated inflammatory response [8,9]. In a meta-analysis of patients with STSS treated with clindamycin, IVIG use was associated with a reduction in mortality from 33.7% to 15.7%; however, the data were derived from one small randomized trial and four non-randomized studies, which substantially limits the strength of evidence [9]. An earlier European randomized trial also suggested possible clinical benefit, but it was terminated prematurely and lacked sufficient statistical power [10]. Therefore, IVIG is not a routine standard of care for every case of STSS, although it may be considered as adjunctive therapy in the most severe cases, particularly in refractory shock and multiorgan failure [5,8]. IVIG was not administered in this case. This decision was based on limited local availability, the limited strength of the available evidence, and the gradual clinical stabilization observed after surgical source control, targeted antimicrobial therapy, and intensive supportive care.

AKI is a common complication of sepsis and septic shock, and sepsis-associated acute kidney injury represents one of the most frequent forms of AKI in patients treated in intensive care units [25,26]. In STSS, rapid development of shock, hypoperfusion, toxin-mediated inflammation, and multiorgan failure may lead to kidney injury and the need for renal replacement therapy [5]. According to the Surviving Sepsis Campaign guidelines, in patients with sepsis or septic shock and AKI requiring renal replacement therapy, both continuous and intermittent modalities may be used; however, CRRT is often selected in hemodynamically unstable patients [7]. In selected patients, CRRT may be performed using adsorptive membranes, which, in addition to renal replacement function, may bind inflammatory mediators and, in the case of some membranes, endotoxin [26]. Current data suggest possible improvement in selected hemodynamic and inflammatory parameters; however, the impact of these techniques on survival and their role in STSS remain uncertain [27,28]. In our case, CRRT was initiated because of AKI during sudden clinical deterioration. Due to suspected STSS and the hyperinflammatory nature of the disease, a heparin-coated AN69ST membrane with adsorptive properties was used. This approach should be considered supportive treatment in a patient who independently met indications for CRRT, rather than an intervention with proven STSS-specific efficacy or proven superiority over other adjunctive therapies.

## 4. Conclusions

This case highlights that even a seemingly minor blunt injury, without disruption of skin integrity, may precede the development of severe invasive GAS infection, NSTI, and clinical features consistent with probable STSS. Given the fulminant course of the disease, a high index of diagnostic suspicion is essential, along with prompt initiation of multimodal treatment, including source control, antimicrobial therapy with an antitoxin effect, and intensive supportive care.

## Figures and Tables

**Figure 1:**
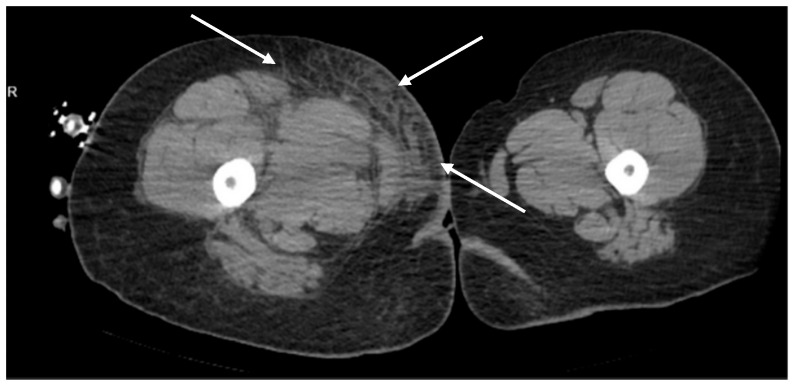
Computed tomography image of the right groin and proximal thigh region showing increased density of the subcutaneous adipose tissue and inflammatory infiltration extending around the iliopsoas muscle toward the posteromedial thigh and the fascia of the upper thigh. Arrows indicate the main areas of inflammatory involvement.

**Table 1:** Laboratory results during hospitalization.

	Reference Values	Admission	Day 2	Day 3	Day 4	Day 7	Day 12	Day 16
WBC, ×10^3^/µL	4.16–11.48	31.23	54.56	68.22	54.91	25.69	9.14	6.86
CRP, mg/L	<5.00	383	453	370	279	31	145	80
PCT, ng/mL	<0.50	19.3	12.5	8.71	5.5	1.34	0.29	0.17
Hb, g/dL	10.8–15.0	12.5	11.7	11.3	11.9	10.3	8.2	9.6
PLT, G/L	162–379	182	187	169	161	93	419	482
Creatinine, mg/dL	0.50–0.90	3.04	1.44	1.18	0.91	0.34	0.42	0.39
Urea, mg/dL	16.6–48.5	69.4	56.4	62.8	55.4	42.4	25.2	25.2
Na^+^, mmol/L	136–146	132	136	140	145	142	134	140
K^+^, mmol/L	3.50–5.10	4.4	3.9	3.4	3.3	3.8	4.1	3.7
AST, U/L	≤35	43	163	145	80	368	24	21
ALT, U/L	≤35	15	53	54	45	310	63	33
Bilirubin, mg/dL	≤1.00	0.28	0.28	0.33	0.43	0.22	0.2	0.17
CK, U/L	<167	532	874	909	–	–	–	–
Myoglobin, ng/mL	25.0–58.0	1066	932	540	–	–	–	–
Lactate, mmol/L	0.56–1.55	8.32	5.33	4.77	4.11	0.89	1.44	0.56

Abbreviations: WBC, white blood cell count; CRP, C-reactive protein; PCT, procalcitonin; Hb, hemoglobin; PLT, platelet count; Na^+^, sodium concentration; K^+^, potassium concentration; AST, aspartate aminotransferase; ALT, alanine aminotransferase; CK, creatine kinase.

**Table 2:** Antimicrobial susceptibility profile of the *Streptococcus pyogenes* isolate. Susceptibility was interpreted according to EUCAST criteria.

Antimicrobial Agent	Interpretation
Ampicillin	Susceptible
Penicillin G	Susceptible
Clindamycin	Susceptible
Erythromycin	Susceptible
Linezolid	Susceptible
Vancomycin	Susceptible

## Data Availability

The data presented in this case report are available from the corresponding author upon reasonable request. Access may be restricted to protect patient privacy.

## Abbreviations

The following abbreviations are used in this manuscript:

AKI	acute kidney injury
AN69ST	acrylonitrile 69 surface-treated membrane
APACHE II	Acute Physiology and Chronic Health Evaluation II
ARDS	acute respiratory distress syndrome
BIA	bioelectrical impedance analysis
CDC	Centers for Disease Control and Prevention
CRRT	continuous renal replacement therapy
CSTE	Council of State and Territorial Epidemiologists
CVVHDF	continuous venovenous hemodiafiltration
EUCAST	European Committee on Antimicrobial Susceptibility Testing
FiO_2_	fraction of inspired oxygen
GAS	group A Streptococcus
ICU	intensive care unit
IVIG	intravenous immunoglobulin
LRINEC	Laboratory Risk Indicator for Necrotizing Fasciitis
MIC	minimum inhibitory concentration
mNUTRIC	modified Nutrition Risk in the Critically Ill score
NSTI	necrotizing soft tissue infection
SOFA	Sequential Organ Failure Assessment
STSS	streptococcal toxic shock syndrome
